# Editorial: Towards the Control of Thermal Expansion: From 1996 to Today

**DOI:** 10.3389/fchem.2019.00284

**Published:** 2019-05-08

**Authors:** Andrea Sanson, Jun Chen

**Affiliations:** ^1^Department of Physics and Astronomy, University of Padua, Padua, Italy; ^2^School of Mathematics and Physics, University of Science and Technology Beijing, Beijing, China

**Keywords:** thermal expansion control, negative thermal expansion (NTE), materials physics and chemistry, materials science, solid state physics

Most materials expand on heating, known as positive thermal expansion (PTE). Thermal expansion is critical in many technological applications, like precise instruments, glazes and coating materials, optical and electronic devices, high-temperature materials design. As a matter of fact, different materials in contact with each other can have different thermal expansion, giving rise to thermal shock breakage. For this reason, controlling thermal expansion represents a challenge for materials design.

Negative thermal expansion (NTE), that is material contraction on heating over a certain temperature range, is relatively rare but has important technological applications. Since the large isotropic NTE was discovered in zirconium tungstate over a wide temperature range in 1996, the interest in NTE has rapidly grown to become the most promising route to achieve the control of thermal expansion. Since then, many NTE materials have been discovered.

NTE phenomenon is known to arise from a range of different physical mechanisms, such as low-energy vibrational modes, ferroelectricity, magnetic, and valence transitions. However, this does not lead to straightforward control of thermal expansion. Moreover, composites of negative and positive thermal expansion materials may fail after repeated cycling, so direct control of thermal expansion within a single homogenous phase is desirable and yet difficult to achieve. To date, different methods are under investigation to achieve the control of thermal expansion.

The present Research Topic includes a collection of original research and review (mini review) articles dedicated to the physical-chemical phenomena connected to NTE and to the state-of-the-art for control thermal expansion. The collection begins with a review by Takenaka on phase-transition type NTE materials, presenting some recent works related to how giant NTE materials are used as practical thermal expansion compensators. Hu et al. present a second review on the NTE behavior in materials with giant magnetocaloric effects (MCE), including some representative examples of MCE materials. An article perspective is presented by Attfield, where NTE is classified in terms of intrinsic structural and electronic mechanisms, and morphological NTE due to the specific morphology of the sample.

Many articles of the collection are dedicated to developing new materials with zero or adjustable thermal expansion. Liu et al. fabricated Zr_2_MoP_2_O_12_/ZrO_2_ composites with a variable coefficient of thermal expansion (CTE) from −5.7 × 10^−6^ K^−1^ to +5.6 × 10^−6^ K^−1^ by changing the Zr_2_MoP_2_O_12_ content, reaching a near zero CTE from 25 to 700°C for the composition ratio Zr_2_MoP_2_O_12_/ZrO_2_ of 2:1. Similarly, Zhang et al. synthetized Zr_2_WP_2_O_12_/ZrO_2_ composites and adjusted the CTE from −3.3 × 10^−6^ K^−1^ to +4.1 × 10^−6^ K^−1^ by varying the mass ratio Zr_2_WP_2_O_12_/ZrO_2_, obtaining again a zero CTE for the ratio 2:1. Near-zero thermal expansion behavior over a wide temperature range was also found out by Li et al. in HfMg_1−x_Zn_x_Mo_3_O_12_ solid solutions, whose phase transition temperature from monoclinic to orthorhombic structure increases with the content of Zn^2+^. Giant NTE properties were observed in Fe-doped MnNiGe compounds by Zhao et al. with CTE values of about −285 × 10^−6^ K^−1^ and −1167 × 10^−6^ K^−1^ in Mn_0.90_Fe_0.10_NiGe and MnNi_0.90_Fe_0.10_Ge, respectively. Furthermore, these materials were combined with Cu in order to control their NTE properties. Guo et al. investigated NTE and magnetic properties of anti-perovskite Ga_1−x_Cr_x_N_0.83_Mn_3_ compounds. They observed that Cr doping introduces ferromagnetic order thus giving rise to the broadening of NTE temperature window. Finally, Zhou et al. obtained huge PTE and NTE in fence-like metal organic frameworks obtained with a series of transition metal ions.

Part of the published works have a computational character. By using an efficient approach for obtaining the variation of the dynamical matrix as a function of the lattice constant, Vila et al. obtained that the large NTE of ZrW_2_O_8_ arises almost exclusively from the transverse contributions of the O-atoms. A second study was performed by Vila et al. on ZrW_2_O_8_, combining theoretical calculations of the structure and vibrational properties with the experimental results from extended x-ray absorption fine structure spectroscopy. *Ab initio* lattice dynamics calculations were combined with inelastic neutron scattering by Singh et al. to study the NTE dynamics in the metal organic framework compound AgC_4_N_3_. Ablitt et al. performed density functional theory simulations in Ruddlesden–Popper layered perovskite series, showing that by changing the fraction of layer interface in the structure one may control the anisotropic compliance necessary for the pronounced uniaxial NTE observed in these systems. Occhialini et al. discussed the broad issue of structural NTE with particular attention on perovskite-structured materials, providing a model treatment beyond mean field theory to identify the key elements that move toward control of NTE. First-principles calculations were performed in NiSi and isostructural compound NiGe by Goel et al. with the aim of studying the phonon modes which give the major contribution to the NTE behavior in NiSi, and reasons for negligible NTE in NiGe.

Lastly, high-pressure synchrotron powder X-ray diffraction studies of Cr_2_Mo_3_O_12_ and Y_2_Mo_3_O_12_ were conducted by Young et al. revealing that the monoclinic polymorphs of these materials do not undergo phase transitions within the studied pressure range, making them unique among the A_2_M_3_O_12_ family of NTE materials. Jiang et al. investigated the thermal expansion properties of the ultraviolet non-linear optical crystal BaAlBO_3_F_2_ and the practical implications were discussed. Wang et al. proposed the new strain gauge method to measure the thermal expansion and magnetostriction of solid materials at low temperatures, opening up an interesting way for exploring potential magnetostrictive and NTE materials.

In summary, the present papers taken together give an overview of research on NTE and on the tuning of thermal expansion. We hope that the different perspectives here reported will inspire new studies and findings toward the design of materials with controlled thermal expansion.

## Author Contributions

All authors listed have made a substantial, direct and intellectual contribution to the work, and approved it for publication.

### Conflict of Interest Statement

The authors declare that the research was conducted in the absence of any commercial or financial relationships that could be construed as a potential conflict of interest.

